# Case Report: Brachydactyly Type A1 Induced by a Novel Variant of in-Frame Insertion in the *IHH* Gene

**DOI:** 10.3389/fgene.2022.814786

**Published:** 2022-05-20

**Authors:** Feier Zeng, Huan Liu, Xuyang Xia, Yang Shu, Wei Cheng, Heng Xu, Geng Yin, Qibing Xie

**Affiliations:** ^1^ Department of Rheumatology and Immunology, West China Hospital, Sichuan University, Chengdu, China; ^2^ State Key Laboratory of Biotherapy and Cancer Center, West China Hospital, Sichuan University, Chengdu, China; ^3^ State Key Laboratory of Biotherapy, Department of Gastrointestinal Surgery, West China Hospital, Chengdu, China; ^4^ State Key Laboratory of Biotherapy, Department of Laboratory Medicine/Research Centre of Clinical Laboratory Medicine, West China Hospital, Chengdu, China

**Keywords:** brachydactyly type A1, IHH, insertion variation, rheumatoid arthritis, anti-cyclic citrullinated peptide

## Abstract

Brachydactyly type A1 (BDA1) is an autosomal dominant inherited disease characterized by the shortness/absence of the middle phalanges, which can be induced by mutations in the Indian hedgehog gene (*IHH*). Rheumatoid arthritis (RA) is a chronic, systemic autoimmune disease characterized by joint destruction, synovitis, and the presence of autoantibodies. In this study, the proband was diagnosed with both BDA1 and RA. We performed whole-exome sequencing in a four-generation Chinese family to investigate their inherited causal mutation to BDA1. A novel in-frame insertion variant in *IHH*: NM_002,181.4: c.383_415dup/p.(R128_H138dup) was identified in the BDA1 pedigree. This insertion of 11 amino acids was located in the highly conserved amino-terminal signaling domain of *IHH* and co-segregated with the disease status. This adds one to the total number of different *IHH* mutations found to cause BDA1. Moreover, we found a potential causal germline variant in *CRY1* for a molecular biomarker of RA (i.e., a high level of anti-cyclic citrullinated peptide). Collectively, we identified novel variants in *IHH* for inherited BDA1, which highlights the important role of this gene in phalange development.

## Introduction

Brachydactyly (BD) is a term referring to disproportionate shortening of fingers and toes and constitutes a heterogeneous group of abnormal development of phalanges, metacarpals, or both (Temtamy and Aglan, 2008) and can be divided into simplex and complex short-finger disease. Simplex includes five clinical types, A, B, C, D, and E, and complex is when there are other symptoms in addition to short fingers, such as facial abnormalities and hypertension. Typically, these include Robinow syndrome, Feingold syndrome, and Temtamy preaxial brachydactyly syndrome (Fitch, 1979; Lyu et al., 2019). BD type A (BDA) is confined to middle phalange shortening, and according to the number and location of affected digits, BDA includes five subtypes (BDA1-5). Except for types A3 and D, whose prevalence is approximately 2%, the other types are all rare diseases (Temtamy and Aglan, 2008). To date, the cause of BD is not fully understood, but simplex BD is mostly inherited in an autosomal dominant manner. Most BD patients will be treated only when it affects functions or for cosmetic reasons.

BD type A1 (BDA1, OMIM: 112500) is an autosomal dominant inherited disease characterized by the shortening or absence of the middle phalanges of most digits (Temtamy and Aglan, 2008). In addition to BDA1, some patients with complex syndromes may have scoliosis, nystagmus, developmental delay, or intellectual disability (Byrnes et al., 2009; Ho et al., 2018). Genetic attribution of BDA1 to *IHH* mutations was first revealed in 2001 (Gao et al., 2001). Since then, increasing genetic evidence has shown that several mutations have been identified in *IHH*, whose protein product plays a critical role in growth, patterns, and morphogenesis (Gao et al., 2001; Giordano et al., 2003; Byrnes et al., 2009; Jang et al., 2015; Ho et al., 2018; Shen et al., 2019; Yang et al., 2020).

*IHH* is an important member of the HH family, which is produced by prehypertrophic chondrocytes. It is related to the formation and differentiation of cartilages and bones (Daoussis et al., 2015; Bechtold et al., 2019; Al-Azab et al., 2020). When the *IHH* gene is mutated, it will weaken the transmission of hedgehog signals, thereby affecting cartilage development. On the other hand, many studies have found that in a variety of arthritis, including rheumatoid arthritis (RA) (Al-Azab et al., 2018), osteoarthritis (Wei et al., 2012), and ankylosing spondylitis (Daoussis et al., 2015), the *IHH* expression level is abnormally elevated. In summary, *IHH* is a key regulator involved in bones and cartilages.

RA is a common chronic autoimmune disease characterized by progressive joint destruction and autoantibodies and has a high disability rate (Weyand and Goronzy, 2021). The mechanism underlying RA is complex, and both genetic and environmental factors can lead to its pathogenesis (Aletaha and Smolen, 2018). In RA, anti-citrullinated protein antibodies (ACPAs) and the rheumatoid factor (RF) have been incorporated into the classification criteria. ACPAs are a key diagnostic indicator of RA and can be positive in the early stage of disease (Van Venrooij et al., 2011). Cyclic citrullinated peptide (CCP) is a test substrate for detecting ACPAs with high specificity and sensitivity. Up to 70% of RA patients are positive for anti-CCP antibodies, and the titer level is highly correlated with the prognosis of RA (Ge and Holmdahl, 2019).

In this study, we investigated the clinical and genetic characteristics of a four-generation Chinese family segregating BDA1. We present herein the clinical and radiographic findings associated with a novel *IHH* insertion variant in 12 affected family members and summarize all BDA1-related *IHH* mutations, expanding the clinical and genetic spectrum of BDA1. Additionally, we aimed to investigate the potential germline variant to the high level of the RA-related indicator (i.e., anti-CCP).

## Materials and Methods

### Subjects

The BDA1-affected family was recruited from West China Hospital for shorted and malformed digits. There were 27 individuals in the BDA1-affected family, including 12 affected individuals. We examined five of these subjects, including bilateral or right radiographs of the hands and feet. Peripheral blood samples were obtained from five affected individuals as well as from three healthy family members. Whole-exome sequencing (WES) was performed on seven individuals of this family, four affected and three normal people. Our study was approved by the Ethics Committee of the West China Hospital, Sichuan University.

### Whole-Exome Sequencing and Variant Calling

Genomic DNA was extracted from peripheral blood using standard protocols of the Blood DNA Mini Kit (Foregene Co., Ltd., Chengdu, China). A minimum of 200 ng of DNA was used for constructing sequencing libraries, which were generated using the Agilent SureSelect Human All Exon V6 kit (Agilent Technologies, CA, United States) following the manufacturer’s recommendations. The captured pools were combined and sequenced on the Illumina Novaseq 6000 platform (2 × 150 bp). Variant calling was performed with a sophisticated pipeline as described previously (Shu et al., 2018; Luo et al., 2021a; Luo et al., 2021b; Chen et al., 2021; Zhang et al., 2021; Liao et al., 2022). Briefly, by using Burrows–Wheeler Aligner (BWA) and GATK4.0 with default parameters, 550 million uniquely mapped reads with MAPQ ≥ 30 were generated, covering 97.97% of target regions at least 30×. A variant allele frequency of 30–70% (variant reads/total reads) was defined as heterozygous, and a variant allele frequency >90% was defined as homozygous. Functional prediction was conducted with SIFT, Polyphen2, and LRT.

### Sanger Sequencing Validation

The variant location was amplified in a single PCR by using primers specific for the region upstream of *IHH* exon 2 (5′-GCG​CCT​ACA​CCT​GCA​CCT​C-3′ and 5′-GGC​GGG​CTC​TTC​ACC​TTC​TC-3′). The candidate *IHH* variant was validated by Sanger sequencing following the standard protocol (Moriyama et al., 2021); alleles were isolated by T-cloning and sequenced by a sequencing primer (5′-CAA​GGA​CCG​CCT​GAA​CTC​GC-3′), and its pathogenicity was classified following ACMG/AMP Guidelines (Richards et al., 2015).

## Results

### Clinical Presentation

**FIGURE 1:**
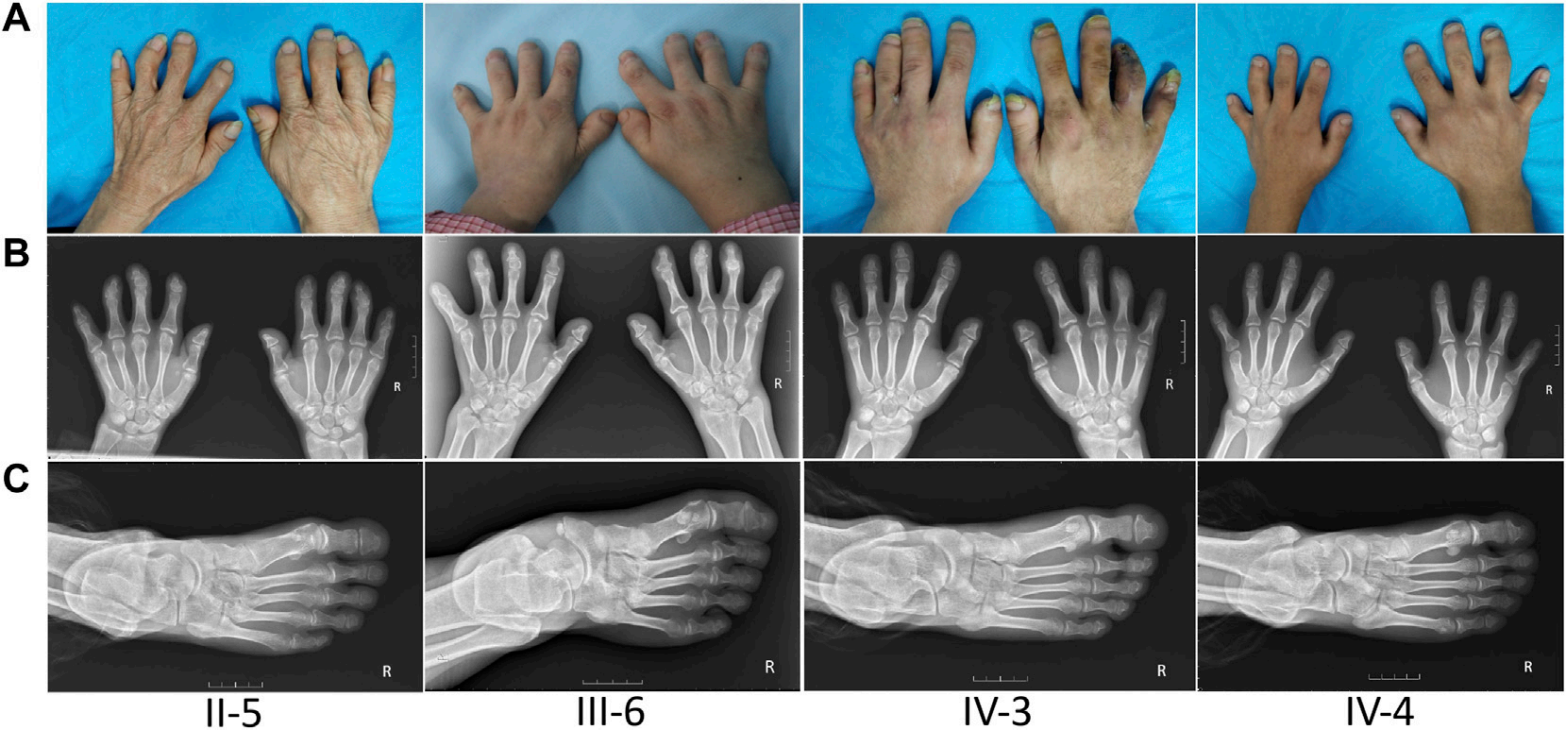
Appearance and radiological findings of other family members with BDA1. **(A)** Shortened fingers and radial deviation of other affected individuals in this family. **(B)** Radiographic images of other patients’ hands: absence of middle phalanges of the second, fourth, and/or fifth fingers (II-5, II-6, IV-3, IV-4) in both hands, and the middle phalanges of the second finger of the right hand were fused to the distal phalanges (IV-4). **(C)** Radiographic images of other patients’ right foot: abnormally shortened toes and the absence of phalanges.

**FIGURE 2:**
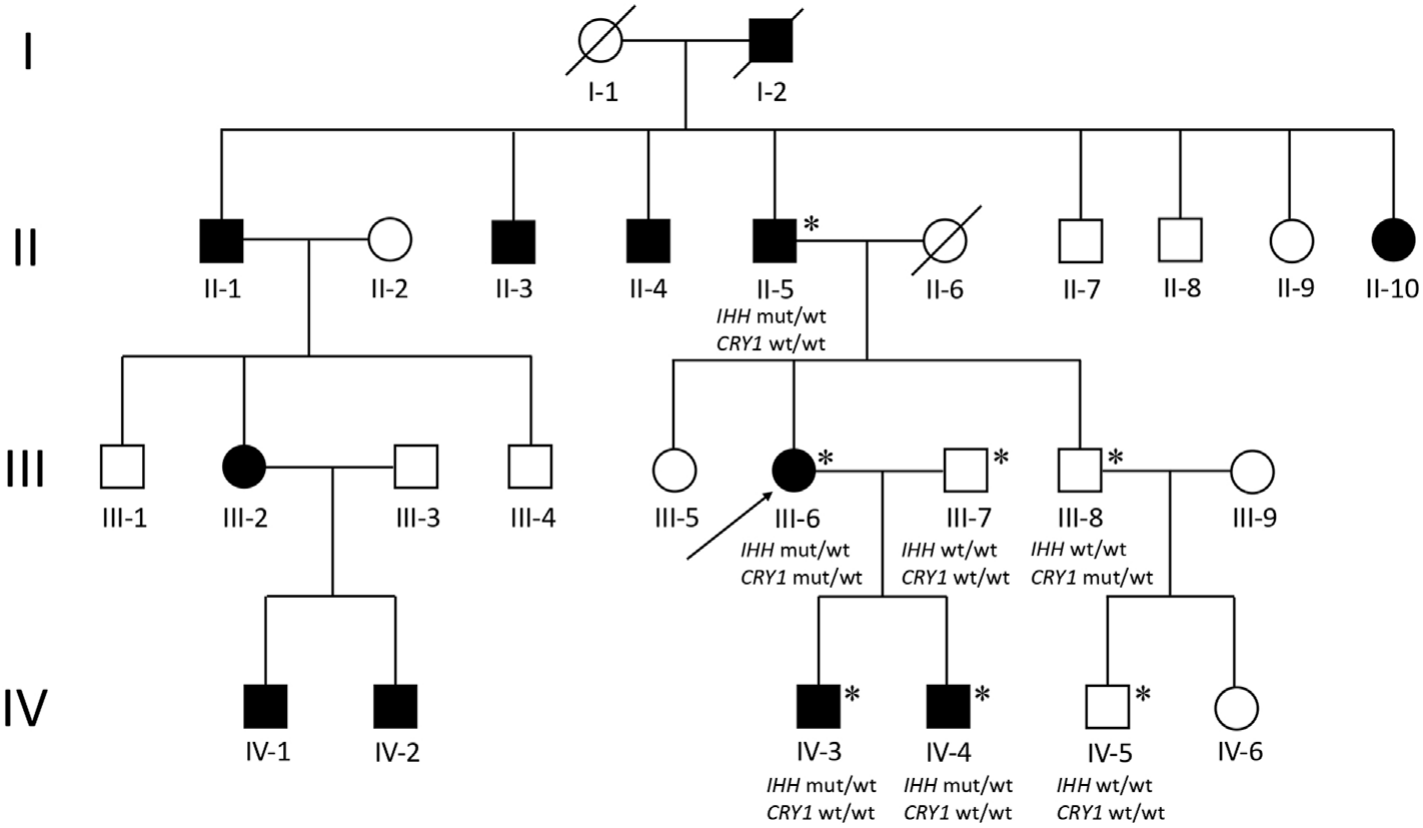
Pedigree of a four-generation family with BDA1. Filled symbols represent affected individuals; open symbols represent unaffected individuals; squares depict males, and circles depict females. The proband is indicated by an arrow. Diagonal lines indicate the proband. Asterisks represent participants who participated in WES testing. Wt, wild type; mut, mutant.

The proband (III-6) was diagnosed with severe RA at the age of 45 years. Upon physical examination, we found that her fingers and toes shortened to varying degrees, and the proximal interphalangeal joints, metacarpophalangeal (MCP) joints, and wrist joints on both sides were swollen and tender. Laboratory tests as the initial presentation showed a high level of RF (49.0 IU/ml, normal: <20), anti-CCP antibody (486 U/ml, normal: <17), immunoglobulin (Ig) G (19.2 g/L, normal: 8.00–15.50), IgA (4,340 mg/L, normal: 836–2900), C reactive protein (CRP) (9.92 mg/L, normal:<5), and positive antibodies, including antinuclear antibodies (ANA) (+1:320, grain type), anti-SSA (++), and anti-Ro-52 (+++) (Table 1). The blood routine test, the urine routine test, hepatic and renal functions, calcium and phosphorous levels in the serum, anti-neutrophil cytoplasmic antibodies, anticardiolipin antibodies, serum C3 and C4 levels, and IgM had normal limits. In addition, X-ray results showed that she had osteoporosis in both hands and feet, bone hyperplasia in hands, the absence of middle phalanges of the second and fifth figures in both hands and of the fourth finger in the left hand, the absence of phalanges of the second to fifth toes in both feet, and shortening of middle phalanges of the third and fourth fingers in the right and of the third finger in left hands; the MCP spaces are uneven across all hands and multiple cystic shadows below the surface of the wrist joint (Figure 1). Therefore, this proband was diagnosed as both BDA1 and RA. Subsequently, we collected her family history and constructed the analysis on the pedigree of the four-generation family for BDA1. Totally, 27 individuals have available information of fingers, including 12 individuals with self-reported shortened fingers (Figure 2). Finally, blood samples were collected from seven family members (four self-reported affected and three normal individuals). After X-ray evaluation, all these four affected individuals manifested shortening of the middle phalanges of all digits. The proband’s father (II-5) showed the absence of the middle phalanges of the bilateral fifth fingers and the second to fifth toes and the shortening of the middle and distal phalanges of the bilateral second to fourth fingers (Figure 1). One of her sons (IV-3) presented with middle phalanges without bilateral fifth fingers and bilateral third to fifth toes, shortening of all distal phalanges of limbs, second to fourth middle phalanges in hands, and second middle phalanges in feet (Figure 1). Her little son (IV-4) exhibited that the fifth middle phalanges in both hands and feet were absent, all distal phalanges of limbs and middle phalanges of the bilateral third and fourth fingers were shortened, and the middle phalanges of the second to fourth toes and the second finger of the right hand were fused to the distal phalanges (Figure 1). In addition, although no clinical signs or symptoms (i.e., joint pain and swelling, morning stiffness) of RA have been self-reported for other family members, the proband’s brother (a nonaffected BDA1 individual, III-8) showed ANA (+1:100, grain type), anti-Ro-52 (+), RF 35.50 IU/ml and anti-CCP >500.0 U/ml (Table 1), which is the molecular sign of RA and is even higher than that of the proband with RA.

**TABLE 1:** Clinical features of tested individuals.

Individual	Gender	Age (y)	Height (±SDS) (cm)	Weight (±SDS) (kg)	Clinical features	Laboratory test	Radiographic features
II-5	Male	70	158 (-1.18)	45.0 (-1.76)	Shortening of fingers and toes	Normal	Absence of middle phalanges of the bilateral fifth fingers and the second to fifth toes and shortening of middle and distal phalanges of the bilateral second to fourth fingers
III-6	Female	45	150 (-0.85)	55.0 (+0.72)	Shortening of fingers and toes; swelling and tenderness of PIP, MCP, and wrist joints on both sides	RF (49.0 IU/ml, normal: <20), anti-CCP (486 U/ml), IgG (19.2 g/L), IgA (4,340 mg/L), CRP (9.92 mg/L), ANA (+1:320, grain type), anti-SSA (++), anti-Ro-52 (+++)	OP in both hands and feet, bone hyperplasia in hands, the absence of middle phalanges of the second and fifth figures in both hands and of the fourth finger in the left hand, and the absence of phalanges of the second to fifth toes in both feet; shortening of middle phalanges of the third and fourth fingers in the right hand and of the third finger in the left hand; MCP spaces are uneven across all hands and multiple cystic shadows below the surface of the wrist joint
III-7	Male	47	175 (+1.81)	65.0 (+1.17)	Normal	Normal	Normal
III-8	Male	48	168 (+0.58)	75.0 (+2.64)	Normal	ANA (+1:100, grain type), anti-Ro-52 (+), RF 35.50 IU/ml, anti-CCP >500.0 U/ml, AKA (+)	Normal
IV-3	Male	22	170 (+0.93)	62.5 (+0.81)	Shortening of fingers and toes, right hand scald	Normal	Middle phalanges without the bilateral fifth fingers and the bilateral third to fifth toes and shortening of all distal phalanges of limbs, the second to fourth middle phalanges in hands, and the second middle phalanges in feet.
IV-4	Male	20	170 (+0.93)	55.0 (-0.29)	Shortening of fingers and toes	Normal	The fifth middle phalanges in both hands and feet were absent, all distal phalanges of the limbs and middle phalanges of the bilateral third and fourth fingers were shortened, and the middle phalanges of the second to fourth toes and the second finger of the right hand were fused to the distal phalanges
IV-5	Male	25	175 (+1.81)	80.0 (+3.38)	Normal	Normal	Normal

SDS, standard deviation score; RF, rheumatoid factor; anti-CCP, anti-cyclic citrullinated peptide; IgA, Immunoglobulin A; ANA, antinuclear antibodies; CRP, C-reactive protein; OP, osteoporosis; MCP, metacarpophalangeal.

### Genetic Analysis

**FIGURE 3:**
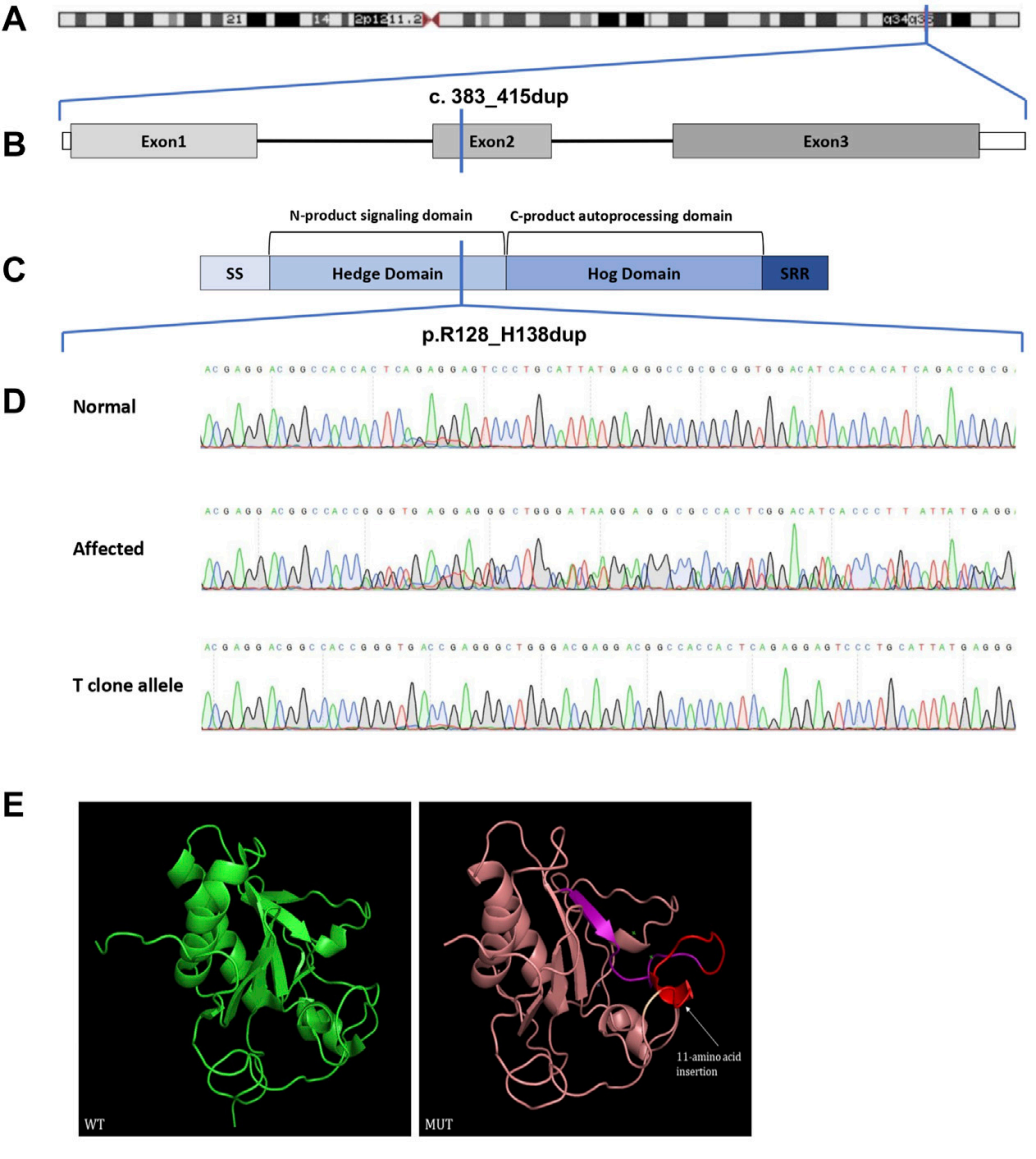
Chromosome location, gene structure, protein structure, and sequence analysis of the Indian Hedgehog. **(A)** Chromosome 2 and the location of *IHH* drawn to scale. **(B)** Genomic DNA map for *IHH*. **(C)** *IHH* protein structure with key domains, regions, and the mutation indicated (SS: signal sequence; SRR: sterol recognition region). **(D)** Sanger sequencing chromatograms. **(E)** Three-dimensional structure modeling of wild-type and mutant proteins. The insert 11-amino acid sequence (red) is same as the forward sequence (purple). WT, wild-type; MUT, mutant.

We performed WES for for BDA1 patients (II-5, III-6, IV-3, and IV-4) and three normal family members (III-7, III-8, and IV-5) to screen out the causal mutation for this BDA1 family (Figure 2). A total of 42,106 nonsilent variants were identified in coding regions or splicing sites. Due to the low prevalence of BDA1, we excluded variants with a minor allele frequency greater than 1% in any ethnic population according to a public database with 141,456 individuals [i.e., gnomAD (Karczewski et al., 2020)], leaving 3,338 variants for further analysis. Given that all the affected individuals carry the variant allele while normal individuals have the wild-type genotype, 28 variants co-segregated with the disease phenotype in this family. We focused on the five variants that are not presented in the gnomAD database (https://gnomad.broadinstitute.org/), including *RAVER2* NM_018,211.4.2:c.C125G/p.(P42R), *PRDM9* NM_020,227.4:c.G979A/p.(E327K), *KRT84* NM_ 033,045.4:c.G263C/p.(G88A), *IFNGR2* NM_ 005,534.4:c.G943A/p.(D315N), and *IHH* NM_002,181.4:c.383_415dup/p.(R128_H138dup) (Figure 3) in all affected family members (Supplementary Table S1). Functional prediction revealed two potential damaging nonsynonymous variants in *PRDM9* and *IFNGR2*. Moreover, the insertion of 11 amino acids in the conserved domain of IHH, which has been validated with Sanger sequencing (Figure 3D), is likely pathogenic (i.e., PM2, PM4, PP1, and PP4) according to the AMP/ACMG guidelines for the interpretation of sequence variants (Richards et al., 2015) and may impact its protein structure (Figure 3E). Intriguingly, previous studies have shown that variants in *IHH* can induce BDA1; thus, we considered the causal status of the *IHH* variant in this BDA1 family.

### Potential Causal Variant for High Anti-CCP Levels

The proband was diagnosed with RA, and her elder brother (III-8, a nonaffected BDA1 individual) also showed ANA (+1:100, grain type), anti-Ro-52 (+), RF 35.50 IU/ml, and anti-CCP >500.0 U/ml. Although this individual exhibited a very high anti-CCP level, which is relatively unique in RA patients, he had no clinical signs or symptoms (i.e., joint pain and swelling, morning stiffness). We considered that this individual may have a high risk of developing RA. Therefore, with the same co-segregation strategy (two affected vs. five unaffected individuals) as BDA1, we identified three germline variant candidates for individuals with abnormal anti-CCP levels in this family. These variants were not present in the gnomAD database and were predicted to be damaging with different methods (i.e., SIFT, Polyphen2, and LRT), including p.I353V in CRY1, p.R119Q in TMEM119, and p.G573D in RBM26 (Supplementary Table S1). No pathogenic history of RA was self-reported from other members, and no available sample for determining anti-CCP levels prevented us from further narrowing down the candidates. However, a few reports have linked *CRY1* to RA, thus making it the top candidate for familial high anti-CCP levels.

## Discussion

Locus 2q35-q36 was identified in response to BDA1 in a previous study (Gao et al., 2001; Kirkpatrick et al., 2003). The *IHH* gene within this locus has not been revealed as one of the causes of BDA1. Since then, a series of variants in *IHH* have been reported to be associated with BDA1 according to HGMD (http://www.hgmd.cf.ac.uk/). In this study, we reported the clinical characterization of four related subjects affected by BDA1. Radiographic investigation of the hands and feet revealed that all four individuals had bilateral generalized shortness of fingers and toes caused by missing or shortened middle phalanges of digits 2–5. By WES, we identified that these subjects are associated with a novel *IHH* insertion mutation p. R128_H138dup, which is the only report.

*IHH* encodes a signaling protein of the Hedgehog family and is essential for bone formation (St-Jacques et al., 1999). *Ihh* knockout mice exhibit embryonic lethality that may be attributed to reduced chondrocyte proliferation, maturation of chondrocytes at inappropriate positions, and failure of osteoblast development in endochondral bones (St-Jacques et al., 1999). The IHH protein contains two major domains, including the Hedgehog amino-terminal signaling domain and the C-product autoprocessing domain, which have proteolytic enzyme activity and cholesterol functions. *IHH* is very conserved among different vertebrates. For instance, it translated into a protein with a length of 411 amino acid residues in both humans and mice, with 95% identity and 97% positivity, supporting its important role in skeletal development in all vertebrates. The majority of reported mutations are located in three hotspots without any truncating variants, including codon 95, 100, and 131, which are adjacent to special locations on the surface of the crystal structure in the conserved Hedge domain (Gao et al., 2001). Consistently, in a mouse model with mutated p.E95K of *IHH*, both *Ihh* ^+/E95K^ and *Ihh* ^E95K/E95K^ mice showed shortened middle phalanges (Gao et al., 2009) compared with normal phalanges in mice that are heterozygous for *Ihh* null mutation heterozygous and embryonic lethality for homozygous *Ihh* knockout mice (St-Jacques et al., 1999). This evidence suggested a dominant effect rather than loss of functions of *IHH* variants. In our study, R128_H138dup includes hot spot 131 and thus may interfere with the interaction of IHH with its binding partners (e.g., PTCH1 and HIP1) (Gao et al., 2001; Gao et al., 2009), according to the predicted protein structure (Figure 3E).

Moreover, two members of this family had very high anti-CCP levels, including the proband who was diagnosed with RA. After filtering the candidate variants with a co-segregation strategy, only *CRY1* out of three candidates was reported to be related to RA. *CRY1* is present in immune and endocrine cells and is expressed in a circadian manner in human cells (Esquifino et al., 2007). When arthritis is induced in a mouse model, *CRY1* knockout mice will experience maximal exacerbation of joint swelling and upregulation of essential cytokines of arthritis, including TNF-α and IL-6 (Hashiramoto et al., 2010). In addition, a positive association of *IHH* expression with RA was noticed in a previous study (Al-Azab et al., 2018). Speculatively, the *CRY1* variant may increase the anti-CCP level, while the combination of *IHH* and *CRY1* variants induces RA development with a high anti-CCP level. However, due to the unavailability of samples from other family members, this hypothesis should be estimated through experimental procedures in the future.

## Data Availability

The datasets for this article are not publicly available due to concerns regarding participant/patient anonymity. Requests to access the datasets should be directed to the corresponding author.
